# Genomics of Chronic Obstructive Pulmonary Disease (COPD); Exploring the SNPs of Protease-Antiprotease Pathway

**DOI:** 10.2174/1389202911314030006

**Published:** 2013-05

**Authors:** Manish Kumar, Neetu Phougat, Sonam Ruhil, Sandeep Dhankhar, Meenakshi Balhara, Anil Kumar Chhillar

**Affiliations:** Centre for Biotechnology, Maharshi Dayanand University, Rohtak, Haryana, India

**Keywords:** Antiprotease, COPD, Inflammation, Metalloprotienases, SNP, TIMP.

## Abstract

The COPD has been an important respiratory condition that affects people worldwide and its incidence has been alarming. The increasing incidence of this disorder has been attributed to global industrialization and environmental pollution. Although the exposures to environmental pollutants and smoking have been important triggers, the genetic component of individuals has been shown to be important for development and progression of COPD. Recent literature reported that protease-antiprotease imbalance to be important in etiopathogenesis of COPD. The enzymes namely neutrophil elastase and matrix metalloprotienases are considered to be foremost proteolytic molecules released by neutrophils and macrophages during inflammatory events in COPD. Normally, the lungs remain protected from the destructive effect of these two antiproteases by α_1_-antitrypsin (α_1_AT) and tissue inhibitors of metalloproteinases (TIMPs) respectively. In this review, we are trying to highlight the work by various research groups in exploring the SNPs of various genes of inflammatory pathways and the protease-antiprotease pathway, which may have some degree of association with COPD.

## INTRODUCTION

Broncho-pulmonary disorders which are mainly associated with allergic manifestations have emerged as an epidemic. The prevalence of these disorders has increased significantly all around the globe. In the developed nations as well as in the developing countries like India, the cases of various broncho-pulmonary disorders such as allergic broncho-pulmonary aspergillosis, bronchiectasis, chronic bronchitis, bronchopulmonary dysplasia, pneumonia, pulmonary fibrosis, asthma and chronic obstructive pulmonary disease (COPD), are enormous. The high rate of prevalence and the cost of treatment, compounded with the loss of man hours, poor quality of life and encumber on care providers have made COPD and asthma the subjects of serious concern among healthcare organizations, policy making government bodies and the scientific communities as well. 

COPD is a general name for the chronic airflow obstruction affecting about 250 million people world-wide. Smoking has been believed to be the dominant cause for the development of COPD; however, recent observations have unveiled the genetic factors associated with this disorder. The patients with COPD require spirometric methods for pulmonary function assessment. The airflow obstruction in spirometry shows a decrease in FEV1 and its ratio to FVC i.e. FEV1/FVC [1, 2]. Although considered irreversible and inexorably progressive, symptoms of COPD may be arrested, or even partially reversed, with early diagnosis, proper treatment, and smoking cessation. Thus both environmental and genetic factors have been found to be involved in etiopathogenesis of COPD. Along with these two major factors, cellular inflammation accompanied by extensive airway remodeling has been the hallmark feature of COPD. Inflammatory events are triggered by the release of selected proinflammatory mediators and various cytokines involved in the recruitment and activation of inflammatory cells such as neutrophils, mast cells and alveolar macrophages (AM). In combination of these biological processes, normal cellular homeostasis is maintained in the body by tightly regulated mechanisms. One such important pathway has been shown to involve cellular proteases and their corresponding antiproteases. The enzymes namely neutrophil elastase (NE) and matrix metalloprotienases (MMPs) are considered to be foremost proteolytic molecules released by neutrophils and AM during inflammatory events in broncho-pulmonary disorders. Normally, the lungs remain protected from the destructive effect of NE and MMPs by α_1_-antitrypsin (α_1_AT) and tissue inhibitors of metalloproteinases (TIMPs) respectively.

The role of *α_1_AT* in COPD has been established with strong supportive evidences, but there is paucity of information on the status of *α_1_AT* genotypes and the association of α_1_AT deficiency with respiratory disorders in Indian population. This remains unclear how the normal allelic variants (PIM1, PIM3) of *α_1_AT* interacts with other genes of antiprotease system in relation to lung homeostasis. Since COPD proceed with complex inflammatory events, *α_1_AT* and *TIMP-1* collectively might play a crucial role in the development and progression of this disorder. Therefore, the present review highlights the outcome of various studies aimed at investigating the genetic polymorphisms and their possible association with predisposition, development and the role of inflammatory biomarkers (cytokines and chemokines) in COPD. This review is focused mainly on protease-antipro tease genes’ SNPs, however the oxidative stress and detoxification genes SNPs are also briefly described.

## CHRONIC OBSTRUCTIVE PULMONARY DISEASE

COPD has been a major health problem and growing cause of mortality across the world. According to the Global Initiative for Lung Disorder (GOLD) estimate, it is likely to become the third leading cause of mortality, worldwide by 2020. The main clinical feature of COPD has been the progressive decline in lung function that is only partly reversible by bronchodilators. Several definitions of COPD have been postulated. The European respiratory society defined COPD as a respiratory disorder which is characterized by airflow obstruction. However, more recent GOLD guidelines defined COPD as a disease with chronic airflow obstruction which is not fully reversible. Chronic bronchitis or the presence of cough and sputum for at least 3 months in each of two consecutive years remains a clinically and epidemiologically useful term. The latter is considered the most appropriate definition of COPD. 

Progression of COPD has been better understood in recent years. The symptoms of COPD such as cough, sputum and dyspnea correlate poorly with pulmonary functions. Airflow obstruction has been documented by spirometry, which showed a decrease in FEV1/FVC below the 5th percentile or less than 70% [3]. The annual decline in FEV1 has been the standard way of assessing the progression of COPD. The smoking has been considered to be the main risk factor for COPD and its cessation is of paramount importance in its management [4]. Despite close epidemiological association of COPD with cigarette smoking, only 10 to 20% of smokers were found to develop disease [5]. The amount of smoking in terms of pack years and duration of smoking, have been estimated to account for only 15% of the variation in FEV1 levels [6]. Hence other factors must contribute to the development of COPD. The childhood viral respiratory infections, latent adenoviral infections and air pollution have been postulated as other environmental risk factors [4]. 

### Prevalence of COPD

COPD has been a major cause of morbidity and mortality worldwide. The study of WHO under Global Burden of Disease Project estimated that COPD was the fifth leading cause of deaths worldwide in 2001 with 250 million patients and it is expected to be the third leading cause by 2020 [7]. Abegunde *et al.* [8] showed that 58 million people died worldwide in 2005, and that 7% of these deaths were caused by chronic respiratory diseases and also 80% (3.1 million) of these occurred in low and middle-income countries. Out of 3.1 million deaths, 1.1 million were younger than 70 years. They have speculated that by 2015 the deaths due to chronic respiratory diseases in low and middle-income countries, including India will be increased upto 4.1 million. Worldwide prevalence of COPD was also studied [9]. 

The incidence of COPD in stage II or more severe cases was 10.1% including 11.8% for men, and 8.5% for women. For those aged 70 years and older, the prevalence was 19-47% for men and 6-33% for women. However it is important to note that in their study, data for prevalence and severity of COPD from Indian subcontinent was not included. Mehrotra *et al.* [10] had reviewed the prevalence of COPD in Africa and observed that there was a deficiency in spirometry based diagnosis of COPD in many African countries which resulted in under reporting of the COPD cases. Buist *et al.* [9] reported that of the total COPD patients in South Africa 22.2% were men and 16.7% were women. The prevalence of COPD in women is reported to be inclined in USA [11]. Stang *et al.* [12] had studied the prevalence of COPD in European countries and reported 2.7, 3, 1.5, 2.6, and 2.6 million cases in Germany, United Kingdom, Spain, Italy and France respectively (Fig. **A**). 

According to NBHLI report, 13.5 million people in USA were reported to suffer from COPD (http://www.wrongdiagnosis.com/c/copd/prevalence-types.html). A stand alone study was conducted by Murthy and Sastry [13] which was related to the impact of COPD on economic fabric of India. This study was based on a total of 14.93 million cases of COPD which included 5.02 million cases of females and 9.92 million males in 2001. Their findings made them to predict a total number of 22.2 million cases of COPD during 2016, in India, which would require an overall cost of rupees 56, 653 million for the treatment of this chronic disorder. This demonstrated the severity in terms of economic burden on healthcare exerted by the COPD [13].

### Pathophysiology of COPD

COPD is characterized by chronic inflammation induced narrowing of the bronchi, resulting in a progressive reduction of airflow and dynamic hyperinflation. Its symptoms include dyspnea, chronic cough, sputum production, and sometimes wheezing. Symptoms may be absent, or ignored by patients, in early disease. Although considered irreversible and inexorably progressive, COPD symptoms may be arrested, or even partially reversed, with early diagnosis, proper treatment, and smoking cessation or decrease. An estimated 25-45% of patients with COPD have never smoked; the burden of non-smoking COPD is therefore much higher than previously believed [14]. It is an inflammatory disease that affects not only all the airways but also the entire body. Systemic presentations of COPD inflammation, particularly the atherosclerosis, are considered to be responsible for most of its associated morbidity and mortality [15]. 

### Role of Inflammation in COPD

There have been numerous studies which have emphasized on multiple factors involved in the progression and pathogenesis of COPD. Orthodox theory on the pathogenesis of COPD suggested that cigarette smoke has been the trigger to activate macrophages, neutrophils and the epithelial cells of the respiratory tract. These cells have been shown to release proteases that degrade tissues and induce oxidative stress in lungs which in turn amplify the activation of transcription factors such as NF-κB that coordinate inflammation via TNF-α and chemokine release. This leads to disease in susceptible smokers, perhaps as a result of inherited mutations in genes and/or in their respective promoters, including those encoding for TNF-α, antiproteases, antioxidants and intrinsic anti-inflammatory molecules such as IL-10. Episodically, the viruses recognized by CD8+ T cells and the bacteria recognized by toll-like receptors (TLR; such as TLR2, 4 and 9) trigger disease exacerbations. Although this theory has been consistent with known findings, it does not explain the persistence of inflammation in smokers who stop smoking.

Histopathological studies on the lung tissues of COPD patients have demonstrated marked inflammation in bronchioles and parenchymal tissue [16]. Such inflammation in airways is thought to contribute directly to the rapid decline in lung function which has been the hallmark feature observed in COPD. The macrophages, neutrophils and CD8+ T cells have been observed to be excessively increased at the sites of parenchymal injury in lungs and also in BAL fluids [17]. In two independent studies by O’Shaughnessy *et al.* [18] and Saetta *et al.* [19] it has been observed that the smokers with COPD had increased numbers of CD8+ lymphocytes as compared the CD4+ cells both in the large and small airways which resulted in decrease in CD4:CD8 ratio. Similar findings were reported in the regional lymph nodes of smokers with COPD, arguing that the CD8+ preponderance extends outside the airway [20]. Similarly one of our previous studies also demonstrated a decrease in the CD4:CD8 ratio in the patients of COPD [21]. Several reports described an imbalance between cytotoxic CD8+ T lymphocytes and helper CD4+ T lymphocytes which may contribute to the abnormal inflammatory process in the airways of patients with COPD to make the disorder a lymphocyte driven inflammatory condition [17, 22, 23]. A low CD4:CD8 ratio has been a characteristic feature of the pulmonary inflammatory response in COPD [17, 19, 24 and 25]. The study of Di Stefano *et al. *[26] showed a decrease in number of CD8+ lymphocytes in the airway biopsies of subjects with severe COPD as compared to control subjects. The CD8+ cells lead to lung damage by secreting cytokines (e.g. TNF-α), which helps in killing the viral infected cells. They also secrete IL-4, IL-5 and IL-13, which may lead to mucus hypersecretion and airway eosinophilia. The exacerbations of COPD are characterized by the influx of eosinophils (and neutrophils) into the lungs, however, the role of T cell derived cytokines in this process has not been yet determined [27, 28].

### Proteolytic Enzymes and Their Association with COPD

Inflammatory cell proteinases are believed to be largely responsible for destruction of the alveolar matrix in COPD and emphysema. The proteinases associated with these inflammatory and immune cells have been divided into three main classes, serine proteinases, MMPs and cysteine proteinases.

### Serine Proteinases

Serine proteinases associated with COPD belong mainly to the SA clan and S1 (trypsin/chymotrypsin) family of proteolytic enzymes. Members of S1 family include NE, proteinase 3 and cathepsin G. These enzymes are synthesized as proenzymes in the endoplasmic reticulum and are processed by cleavage of the signal peptide and removal of a dipeptide by cathepsin C which then gets stored in granules as active packaged protein. The proteinases have also been reported to be present in the subsets of peripheral blood monocytes. Other immune cells, such as mast cells, also contain significant amounts of serine proteinases. The NE is a potent secretagogue and the resultant mucus might aggravate airflow obstruction in COPD. However, the main role is related to its capacity of causing alveolar destruction. The elastase-antielastase hypothesis regarding pathogenesis of COPD strongly described the role of NE in respiratory disorders. This hypothesis was built upon two seminal observations. Firstly, the instillation of elastases resulted in emphysema [29] and secondly, the patients with deficiency of α_1_AT, the endogenous inhibitor of NE, were found to be at increased risk of developing disease [30]. The role of NE in COPD and emphysema was studied using experimental animals. The exposure to any COPD trigger in mice deficient for NE [31] resulted in only 40% as much airway constriction as wild-type mice suggesting its importance in progression of COPD and emphysema. 

### Matrix Metalloproteinases

MMPs are a family of 26 matrix degrading enzymes which collectively degenerate all of the protein components of the extracellular matrix [32]. They are believed to be essential for the development, tissue remodeling and repair. MMPs are produced by a range of stromal cells and by two of the major inflammatory cells i.e. neutrophils and AM which play central role in pulmonary disorders [33, 34]. They can be distinguished from other endopeptidases by their dependence on metal ions as cofactors, to degrade extracellular matrix and were first described in vertebrates [35], including *Homo sapiens*, but have since been found in invertebrates and plants also. Initially, the enzymatic activity (collagen triple helix degradation) was observed during tadpole tail metamorphosis. Therefore, the enzyme was named as interstitial collagenase which was later called MMP-1. The members of MMP family have been found to share 40-50% identity at the amino acid sequence level and possess common structural domains. The abnormal expression of MMPs has been found to be associated with many destructive processes including tumour cell proliferation, arthritis, atherosclerosis, arterial aneurysms and pulmonary emphysema. The members of the MMP family were originally identified by descriptive names that were assigned on based membrane type and limited knowledge of their preferred substrate specificities e.g., collagenases, gelatinases, stromelysins and matrilysins [36]. The MMPs could be divided into subgroups according to their primary substrates like collagenases (MMP-1 and MMP-13) that degraded interstitial collagens, gelatinases (MMP-2 and MMP-9) that degraded BM components like fibronectin and elastin and the stromelysins (MMP-3, MMP-10 and MMP-11) that have a broad spectrum of extra cellular membrane (ECM) targets. The so-called ‘membrane-type’ (MT) MMPs form a distinct group, since they were found to bind the cell membrane and were rarely inhibited by tissue inhibitor proteinases like TIMP-1. Sputum MMP-12 concentrations have been found to be greater in patients with COPD and smokers [37] and MMP-12 SNPs have been associated with COPD [38].

### Role of MMP-9 in COPD

MMP-9 (92-96 kD gelatinase B) has been a member of gelatinase subfamily and considered to be involved in the cellular invasion of the basement membrane by cells (e.g. T cells, mononuclear phagocytes, synovial fibroblasts, and metastatic tumor cells) involved in arthritis and cancer [39-41]. The sources of MMP-9 have been keratinocytes, monocytes, leukocytes, macrophages, and fibroblasts and they have a gelatin-binding domain [42]. In common with all MMPs, the gelatinases are known to be produced in a latent form requiring activation to produce their enzymatic effects. The substrates for MMP-9 include type IV collagen in basement membrane [43]. In COPD, MMP-9 has been considered to play a major role in cell migration and airway inflammatory responses. Increase in proteolytic activity is expected during acute airway eosinophilic inflammation. *In vitro* and* in vivo* studies [44] also suggested that MMP-9 may play an important role during eosinophil migration. The association of MMP-9 with other diseases such as chronic inflammatory autoimmune diseases, including rheumatoid arthritis, Sjögren’s syndrome, idiopathic uveitis and systemic lupus erythematosus has also been reported [45-47]. An acute increase of 10 to 160 fold in the concentrations of MMP-9 and MMP-3 in the epithelial lining fluid of asthma patients undergoing mechanical ventilation was reported [48]. The MMP-9 could trigger inflammation directly, by tissue destruction, or indirectly, by generation of inflammatory signals and also by recruitment of inflammatory cells [49]. These studies highlighted the importance of role of MMP-9 in COPD related airway remodeling.

It is known that the turnover and remodeling of ECM have been tightly regulated. The uncontrolled proteolysis of ECM by MMP-9 or excess collagen deposition due to their impaired activity may be associated with structural changes in the airways. The activity of MMP-9 in the body is controlled by specific inhibitor, called TIMP-1 [50]. 

### Role of MMPs and TIMPs in Bronchial Disorders

MMPs and their inhibitors are significant contributors to the pathogenesis of COPD via their influence on the function and migration of inflammatory cells as well as matrix deposition and degradation. Thus, an increase in the molar ratio of MMP/TIMP would favor tissue injury, while the reverse could be associated with increased fibrosis. 

Though it has been clear that enhanced airway inflammation in COPD may be associated with increased expression of MMPs [51], whether specific inhibitors of MMPs could reduce airway injury and facilitate orderly healing in COPD, details of this mechanism is still unknown. As MMP-9 plays a role in tissue remodeling during physiologic and pathologic processes by initiating the degradation of the ECM, the over expression of MMPs might lead to tissue destruction that is characteristic of chronic inflammatory diseases such as COPD, rheumatoid arthritis and scleritis [52, 53]. Lung macrophages are found to release elastinolytic enzymes, including MMP-9, along with tissue inhibitor of MMPs, AMs of COPD patients released greater amounts of MMP-9 with high enzymatic activity than the normal individuals. As a result there has been degradation of elastin in the walls of the alveoli, leading to the functional destruction of these organs. Subsequently the bronchioles collapse to obstruct the flow of air. 

In contrast, AM of normal individuals, release more amount of TIMP-1 than the AM of COPD patients. The LPS and IL-1β have been responsible for a dose-dependent increase in MMP-9 release and also for its activity, together with increased levels of TIMP-1 [54]. 

### Proteases-Antiproteases Imbalance: A Key Phenomenon in COPD

COPD involve extensive remodeling of lung tissue structure and hence protease-antiprotease imbalance has been implicated to play a significant role in the pathogenesis of bronchial disorders. NE and MMPs are prominent proteolytic molecules released by neutrophils and AM during the inflammatory events. NE is a protease which is capable of destroying major structural proteins of the alveolar wall. The α_1_AT is primarily synthesized by hepatocytes and has been responsible for inhibition of NE in the lungs, thus maintaining the lung structure and integrity by keeping a check on NE activity. The MMPs have been reported to play a major role in regulating cell behaviors such as cell proliferation, migration (adhesion/dispersion), differentiation, angiogenesis, apoptosis and host defense. The concentration of MMP-9 is kept under tight control by its respective inhibitor TIMP-1. 

## GENETIC STUDIES IN COPD

COPD like asthma can be described as a complex human disorder influenced by multiple genes, environmental factors and gene-by-environment interactions. The genes that contribute to the development of COPD may do so via several different mechanisms. The environmental and genetic factors have been considered to be responsible for the familial occurrence of COPD. Several investigators have shown increased prevalence of COPD in the relatives of cases, compared with the prevalence of COPD in relatives of controls [55-57]. The increased prevalence could not be explained by differences in other known risk factors. In addition, there is a higher correlation of lung function between parents and children or between siblings than between spouses [58, 59]. The prevalence of COPD and similarity in impaired functions of lungs was found to decrease with increased genetic distance [55, 60, 61]. However, this has not provided definitive evidences for the existence of genetic risk factors for COPD. Studies related to twin subjects have provided more reliable means of determining the genetic contribution to inheritance of lung function. Relative importances of genetic and environmental effects have been assessed on the monozygotic and dizygotic twins [60, 62-67]. The pattern of inheritance of pulmonary functions in families, therefore, has been suggested to be important for drawing inferences concerning involvement of genetic components in COPD. This approach is known as segregation analysis and the results of such studies have confirmed a significant genetic contribution in pulmonary function [68-71]. However, the results of most of these studies have indicated that the genetic component is composed of several genes, each with a limited effect on the functional efficiency of the lungs.

### Candidate Genes for COPD

COPD has been characterized by a slowly progressive irreversible airflow obstruction which is due to peripheral airway inflammation and loss of lung elastic recoil resulting from parenchymal destruction. Many inflammatory cells, mediators and enzymes are found to be involved, but their relative importance is still poorly established. As discussed previously, COPD can be considered as a complex disorder which is mediated by interaction between the genes and environmental factors. Till now only few genes have been reported to be associated with the pathogenesis of COPD. These genes are found to be mainly involved in antiproteolysis, metabolism of toxic substances in cigarette smoke, AHR and the inflammatory response to cigarette smoke. The candidate genes and their polymorphisms potentially involved in the pathogenesis of COPD are described below.

### Genes of Inflammatory Pathway in COPD

Genetic studies in COPD have mainly dealt with inflammatory mediators and are of significant value in understanding its pathophysiology. Some of them are discussed in this section. The role of gene responsible for the synthesis of Vitamin D-binding protein (VDBP) in pathogenesis of COPD has been indicated. The VDBP has been found to exist in three major isoforms 1S, 1F and GC2. The individuals who had one or two copies of allele 2 (GC) were found to be protected against COPD [72] and those with homozygous 1F were at increased risk of developing COPD. *TNF-α* and *TNF-β* genes also have been considered to play important role in the pathogenesis of COPD. Several polymorphic forms of these genes have been reported such as G-308A in the *TNF-α* gene promoter and A252G in first intron of *TNF-β* gene. These polymorphisms have been found to be associated with the level of TNF-α and TNF-β production *in vitro *[73]. The -308A allele of *TNF-α* has been reported to be associated with cerebral malaria, asthma and chronic bronchitis also [74-77]. However, there are some studies which have not shown any association of this allele with the prevalence of COPD [61, 78, 79]. The genes of interleukins belonging to IL-1 family also have been studied for their role in the progression of respiratory diseases including COPD. IL-1α and IL-1β are two important pro-inflammatory cytokines whereas IL-1 receptor antagonist (IL1RN) is a naturally occurring anti-inflammatory agent. There was a SNP (C511T) reported in the promoter region of *IL-1β* gene and a penta-allelic polymorphism in intron 2 of *IL1RN* gene [80, 81]. It has been reported that the allele 2 of *IL1RN* gene is associated with chronic inflammatory diseases such as ulcerative colitis, systemic lupus erythematosus and alopecia areata [82-85]. Joos *et al. *[86] did not find any association of *IL-1* genotypes with the rate of decline of lung function in smokers, however, they observed a significant contribution of *IL1RN/IL1-β* haplotypes in these individuals. Similarly Ishii *et al. *[87] did not find any association of *IL-1β* and *IL1RN* polymorphisms with COPD in a Japanese population.

### Genes of Oxidative Pathway in COPD

Gene expression analysis using Affymetrix arrays revealed mRNAs representing 341 out of 642 oxidative stress genes from two predefined gene sets to be differentially expressed in healthy nonsmokers when compared with healthy smokers, and 200 differentially expressed oxidative genes in subjects with COPD when compared with healthy smokers [88]. The *CYP2E1* and *NAT2* variants have been reported to be associated with COPD in Indian subjects [89].

### Genes of Proteases-Antiprotease Pathway in COPD

Intricate balance between proteases and antiproteases has been reported to play a crucial role in regulation and maintenance of tissue integrity of lungs in COPD. The COPD progresses through disruption of lung epithelium and subsequent influx of various inflammatory mediators. Amongst the numerous molecules involved in these pathways, proteases (NE and MMPs), and their inhibitors (α_1_AT and TIMPs) are considered to play significant roles. The α_1_AT has been an acute phase protein synthesized in the liver. The MM allele of *α_1_AT* gene has been reported to be the wild type allele which expresses normal levels of α_1_AT. The two common variants of *α_1_AT*, the Z and S are resulted from point mutation in *α_1_AT* gene [90]. The individuals with ZZ genotype have been found to have 100% risk for developing COPD. Despite the strong association of ZZ genotype with early-onset of COPD, the clinical course of the disease has been observed to be highly variable [91]. Therefore, the contribution of ZZ genotype in increasing the risk of lung function impairment is likely to be overestimated due to selection bias. It may be possible that other genetic factors influence the clinical course in ZZ homozygotes. The common cause of intermediate deficiency of α_1_AT is MZ or MS heterozygosity, present in 3-10% of Caucasian populations. The MZ and MS heterozygotes show 60-80% reduction in α_1_AT levels as compared to normals. The occurrence of SZ heterozygotes has been rare but the individuals with this allele may have levels of α_1_AT even lower than 40% of normal thus making heterozygotes to be at increased risk for COPD if they are smokers [92]. Contrary to these observations, a study from Spain showed no association between SZ phenotype and COPD [93]. The results of many case-control studies have shown an increased prevalence of the *αMZ* heterozygotes in COPD patients with respect to controls [61, 94-98].

There are some polymorphic forms of *α_1_AT* gene that did not show any association with α_1_AT deficiency but showed association with COPD in some populations [99, 100]. However, their association with COPD in other populations could not be confirmed [61, 101]. The genetic defects in *α_1_AT *have been reported to be associated with emphysemic damage in COPD*.* In another case-control study by Kwok *et al. *[102] in a Chinese population, PIZ allele was not detected and there was no significant difference in distribution of PIM phenotypes/subtypes between patients with COPD and healthy controls. There was also a significant difference in the proportion of variant S and F alleles between the disease group and the control population. In one of our earlier studies [103] the frequency of the PIM3 allele in COPD patients was found to be significantly higher than the controls (P = 0.0001). Five SNPs, including a novel SNP (24_25insA), were observed near the junction of exon-intron-I. The occurrence of these SNPs didn’t show any association with COPD. PIM3 was also present in 38% normal individuals with no symptoms of COPD. This observation emphasized the search for genetic abnormalities of other proteases and antiproteases that may be involved in lung destruction. α_1_ACT is another protease inhibitor which is secreted by the liver and AM. Several polymorphic forms of *α_1_ACT* have been shown to be associated with COPD [100, 104], however, some other investigators found no such association [101, 105]. The alpha-2-macroglobulin (α_2_M) is a broad spectrum protease inhibitor which is also synthesized in hepatocytes and in AM. At least three variants of the *α_2_M* gene have been described which may predispose the host to develop chronic lung disease [100]. There is very little information regarding the genetic role of various TIMPs in pathophysiology of COPD. However, several promoter polymorphisms in *MMP* genes were shown to alter gene expression and possibly the lung function [44, 107-109]. A recent study found that haplotypes consisting of alleles from the *MMP-1 *G-1607 GG and *MMP-12 *Asn 357 Ser polymorphisms were associated with rate of decline of lung function. The data suggested that polymorphisms in the *MMP-1* and *MMP-12* genes are either causative factors in smoking related lung injury or are in linkage disequilibrium with causative polymorphisms [110]. Overall, these polymorphisms are rare and the evidences supporting their association with susceptibility to COPD has been less convincing.

We performed detailed genotyping studies on patients and controls for *TIMP-1* gene (Fig. **C**). Results led to the identification of seven polymorphisms, of which six namely, rs5953060 in intron-IV, rs4898 (Phe124Phe) in exon-V, rs6609533 in intron-V, rs11551797 (Ile158Ile) in exon-VI, rs2070584 and rs6609534 in 3’ region were already reported. SNPs Phe124Phe and Ile158Ile of *TIMP-1* gene were not found to be associated with COPD in Indian population [103]. However, Diemen *et al.* reported that the SNPs Phe124Phe and Ile158Ile were associated to decline in FEV_1 _in male Caucasian COPD subjects [111]. An additional novel polymorphism (G to A) of *TIMP-1* gene was located in the intron IV at base position 3774. An intronic polymorphism of *TIMP-1* (rs6609533) with G/A transition was found to be associated with COPD [103]. Serum concentration of TIMP-1 in patients with wild type genotype did not differ significantly from the patients with mutant genotypes. Therefore we concluded that this intronic SNP (rs6609533) did not have any effect on the serum level of TIMP-1. The allele frequency of intronic SNP (rs6609533) of *TIMP-1* gene was significantly higher in COPD patients than the controls. Therefore, we investigated whether this intronic SNP (rs6609533) of *TIMP-1* interacted with PIM3 of *α_1_AT* in COPD. The analysis of the interaction of above mentioned two SNPs showed high epistatic association among *α_1_AT* and *TIMP-1* genes. In view of these observations we considered that PIM3 of *α_1_AT* and rs6609533 of *TIMP-1* gene could be important genetic markers for use in better management of COPD.

## Figures and Tables

**Fig. (A):**
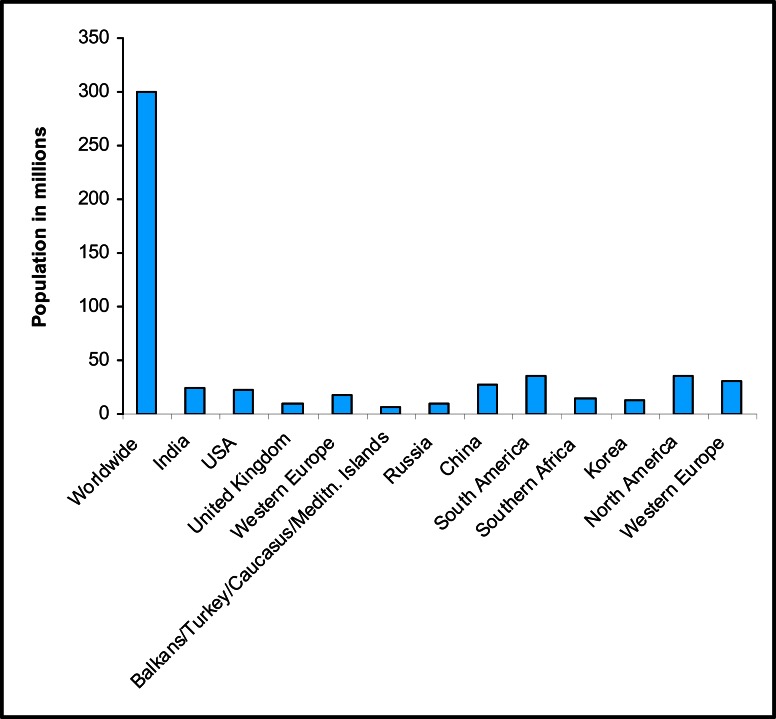
Prevalence of COPD in different countries around the world.

**Fig. (B):**
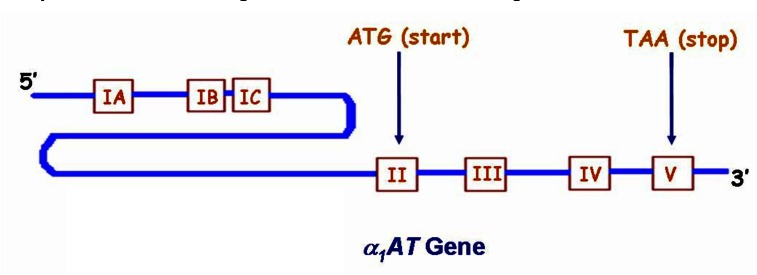
The structure of *α_1_AT* gene showing all the exons.

**Fig. (C):**
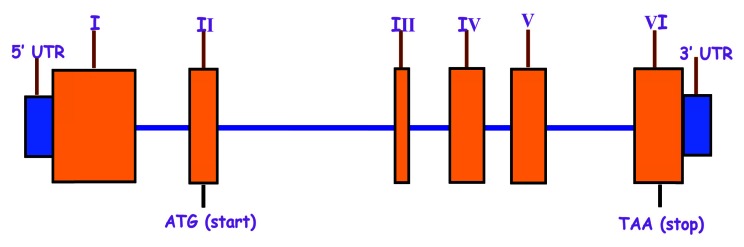
The structure of *TIMP-1* gene showing the all the exons.

## CONCLUSION AND FUTURE DIRECTIONS

COPD is a major respiratory disorder found all over the world and their prevalence has risen to alarming proportions in India as well. The results of the present study have confirmed the association of PIM3 mutation in *α_1_AT* gene with development of COPD. The analysis further indicated that PIM3 alone may not be enough to exert deleterious effects in the host, instead it becomes pathogenic in combination with rs6609533 genotype of *TIMP-1*. The antiprotease, TIMP-1, which is reported to play a potential role in the maintenance of protease/antiprotease balance in the pulmonary tissues, was found to be crossly related to the progression of the respiratory disorders. In light of the progress made in the field of pharmacogenomics and toxicogenomics it is expected that the SNPs of the protease-antiprotease pathway will be screened functionally and will give a direction to more individualized treatment for this disorder.
